# Paucity of qualitative research in general medical and health services and policy research journals: analysis of publication rates

**DOI:** 10.1186/1472-6963-11-268

**Published:** 2011-10-12

**Authors:** Anna R Gagliardi, Mark J Dobrow

**Affiliations:** 1 Departments of Surgery; and Department of Health Policy, Management and Evaluation; and Institute of Medical Science, Faculty of Medicine, University of Toronto, Toronto, Ontario, Canada; 2 Cancer Services and Policy Research Unit, Cancer Care Ontario, Department of Health Policy, Management and Evaluation, University of Toronto,Toronto, Ontario, Canada

## Abstract

**Background:**

Qualitative research has the potential to inform and improve health care decisions but a study based on one year of publications suggests that it is not published in prominent health care journals. A more detailed, longitudinal analysis of its availability is needed. The purpose of this study was to identify, count and compare the number of qualitative and non-qualitative research studies published in high impact health care journals, and explore trends in these data over the last decade.

**Methods:**

A bibliometric approach was used to identify and quantify qualitative articles published in 20 top general medical and health services and policy research journals from 1999 to 2008. Eligible journals were selected based on performance in four different ranking systems reported in the 2008 ISI Journal Citation Reports. Qualitative and non-qualitative research published in these journals were identified by searching MEDLINE, and validated by hand-searching tables of contents for four journals.

**Results:**

The total number of qualitative research articles published during 1999 to 2008 in ten general medical journals ranged from 0 to 41, and in ten health services and policy research journals from 0 to 39. Over this period the percentage of empirical research articles that were qualitative ranged from 0% to 0.6% for the general medical journals, and 0% to 6.4% for the health services and policy research journals.

**Conclusions:**

This analysis suggests that qualitative research it is rarely published in high impact general medical and health services and policy research journals. The factors that contribute to this persistent marginalization need to be better understood.

## Background

Historically, quantitative research has been the most sought after evidence to support health care decision making by clinicians, managers and policy makers, henceforth referred to as users. However, many important questions are not easily answered by quantitative methods, and decisions may be sub-optimal in complex health care environments where quantitative data alone do not address varied information needs [1]. Users also require contextual information about the feasibility and appropriateness of interventions, data that could be supplied by qualitative research [2-6]. Qualitative methods allow complex issues to be studied, and can produce rich data on perceptions, beliefs, experiences and behavior to create a thorough understanding of a problem, and how it could be resolved [7]. Qualitative research approaches have been used to improve health service delivery for a variety of clinical conditions and settings [8-14].

Long characterized as anecdotal or subject to biases, qualitative research has had a much lower profile than quantitative research in health care decision making [5,6]. This may be partly related to the observation that few qualitative studies appear to be published in major health care journals, which remain a primary means of disseminating research. For example, McKibbon's analysis of qualitative studies published in clinical journals during the year 2000 showed that 0.6% of research articles published in 170 general medical, mental health and nursing journals reviewed were qualitative [15]. Their study found that the majority (61.0%) of qualitative studies were published in 17 nursing journals, while few were published in what were considered high impact journals. They also noted that four of the top 20 journals for the year 2000 published 15 qualitative studies, of which 12 were published in the BMJ.

Interpretation of these findings is limited because they were based on studies published during a single year in a convenience sample of journals articles that had been assembled for a specific research project. While some assert that interest in qualitative research is on the rise, it is unclear whether publication rates of qualitative research in prominent sources have similarly risen [16,17]. A more detailed, longitudinal analysis of the availability of qualitative research in top health care journals is needed. The purpose of our study was to explore whether qualitative research publication rates increased over a ten year period from 1999 to 2008 in health care journals sampled based on their impact in the international scientific community.

## Methods

### Approach

A literature search was conducted to identify and count the number of qualitative research articles compared with the total number of research articles published in top ranked health care journals over the last decade, including general medical, and health services and policy research journals. Ethics approval was not required since analysis was based on publicly available data.

### Sampling

The most frequently top ranked ten general medical, and ten health services and policy research journals were identified based on performance across four ranking systems reported in the 2008 ISI Journal Citation Reports (two-year and five-year impact factor, Eigenfactor, ArticleInfluence). We chose the ten most highly ranked journals across the four systems for general medical and health services and policy journals since they each generate variable ranking, and there is no consensus on which approach is most accurate [18]. Eligible journals are presented in Table 1.

**Table 1:** Selected general medical, and health services and policy research journals

General medical*	Health services and policy research*
American Journal of Medicine	American Journal of Managed Care
Annals of Internal Medicine	Health Affairs
Archives of Internal Medicine	Health Economics
BMJ CMAJ	Health Services Research Journal of Health Economics
Journal of the American Medical Association	Medical Care
Journal of Internal Medicine	Medical Decision Making
Lancet	Milbank Quarterly
New England Journal of Medicine	Quality & Safety in Health Care
PLOS Medicine	Value in Health

*in alphabetical order

### Data collection

MEDLINE was searched to identify the total number of qualitative articles published in each of the 20 eligible journals over the period from 1999 to 2008 using the strategy in Table 2. Search strategies for identifying qualitative research in MEDINE have been developed [19]. Others have shown that searching for qualitative research in MEDLINE involves trade-offs between recall and precision [20,21]. That study found that even the search with the highest recall resulted in poor precision, with 96% of identified items deemed irrelevant. Therefore we opted to use a simple search strategy, assuming that limitations in accurately identifying qualitative research applied equally to each journal.

**Table 2:** Search strategy used to identify qualitative articles published in eligible journals during 1999 to 2008

Data element	Search strategy
Numerator (total number of empirical qualitative articles published)	journal title AND (qualitative research OR interviews as topic OR focus groups) NOT (comment OR editorial OR letter OR news)
Denominator (total number of empirical research articles published)	journal title NOT (comment OR editorial OR letter OR news)

Two authors (ARG, MJD) independently reviewed titles and abstracts to identify qualitative research studies. Eligible studies included program evaluations, case studies, interviews, focus groups, content analysis of documents or discourse analysis, or field observation focused on any type of policy, management or clinical aspect of health care delivery or organization; which explored, described or compared knowledge, attitudes, beliefs, opinions, views, experiences, behaviour, practices, and contexts or environmental factors influencing any of these issues; and provided methodological details of sampling, recruitment and analysis. Ineligible studies included randomized controlled trials or clinical cohort studies that may have incorporated a qualitative component, interviews involving time trade off choices or close-ended questionnaires, or studies where interviews were conducted to develop questionnaire content but emphasis of the study was on reporting of psychometric testing with little or no qualitative methodologic details, and narrative systematic reviews. Independent selections were tabulated, and full-text articles were retrieved and reviewed to resolve discrepancies.

To validate our findings, additional checks were performed. Tables of contents (or abstract or methods section of article if necessary) were scanned across two different years for two general medical (CMAJ 2001, BMJ 2006) and two health services and policy research journals (Medical Care 2001, Health Affairs 2006) to quantify the number of qualitative and non-qualitative studies, and compare these data with the MEDLINE results. One author independently reviewed selections of qualitative studies made by a research assistant from tables of contents for the four journals.

### Data analysis

The number of qualitative and non-qualitative research studies identified in MEDLINE and tables of contents were quantified, and the percentage of total studies that were qualitative calculated per journal per year over the ten year period from 1999 to 2008. This data was scanned to identify changes but trends were not analyzed statistically.

## Results

Tables 3 and 4 show the number of qualitative and total studies identified by the literature search, and the percentage of qualitative studies for general medical and health services and policy research journals, respectively. The total number of qualitative studies published in general medical journals from 1999 to 2008 ranged from 0 in the *Journal of Internal Medicine *and *New England Journal of Medicine *to 41 in the *BMJ*. The percentage of qualitative studies in general medical journals over this period ranged from 0.0% to 0.6%. No trends in the number of qualitative studies published yearly were apparent except for a small peak in 2002 for the *BMJ*.

**Table 3:** Number/percentage of qualitative articles published in top general medical journals, 1999 to 2008

Journal	1999	2000	2001	2002	2003	2004	2005	2006	2007	2008	Total
Am J Med											

qualitative empirical articles	0	1	0	0	0	1	2	1	0	0	**5**

total empirical articles	287	242	303	267	267	299	326	306	264	259	**2820**

**percentage qualitative**	**0.0**	**0.4**	**0.0**	**0.0**	**0.0**	**0.3**	**0.6**	**0.3**	**0.0**	**0.0**	**0.2**

Ann Intern Med											

qualitative empirical articles	2	1	0	0	0	1	0	0	1	2	**7**

total empirical articles	261	242	308	308	315	267	296	282	266	255	**2800**

**percentage qualitative**	**0.8**	**0.4**	**0.0**	**0.0**	**0.0**	**0.4**	**0.0**	**0.0**	**0.4**	**0.8**	**0.3**

Arch Intern Med											

qualitative empirical articles	1	1	1	1	0	1	3	0	1	0	**9**

total empirical articles	278	359	335	309	301	302	312	288	280	261	**3025**

**percentage qualitative**	**0.4**	**0.3**	**0.3**	**0.3**	**0.0**	**0.3**	**1.0**	**0.0**	**0.4**	**0.0**	**0.3**

BMJ											

qualitative empirical articles	2	3	8	14	7	2	2	1	0	2	**41**

total empirical articles	677	624	592	676	754	622	600	534	565	695	**6339**

**percentage qualitative**	**0.3**	**0.5**	**1.4**	**2.1**	**0.9**	**0.3**	**0.3**	**0.2**	**0.0**	**0.3**	**0.6**

CMAJ											

qualitative empirical articles	0	2	2	2	0	0	0	0	0	0	**6**

total empirical articles	268	241	223	247	251	237	266	227	212	206	**2378**

**percentage qualitative**	**0.0**	**0.8**	**0.9**	**0.8**	**0.0**	**0.0**	**0.0**	**0.0**	**0.0**	**0.0**	**0.3**

J Intern Med											

qualitative empirical articles	0	0	0	0	0	0	0	0	0	0	**0**

total empirical articles	146	151	118	124	142	136	121	124	123	110	**1295**

**percentage qualitative**	**0.0**	**0.0**	**0.0**	**0.0**	**0.0**	**0.0**	**0.0**	**0.0**	**0.0**	**0.0**	**0.0**

JAMA											

qualitative empirical articles	2	0	0	0	2	0	0	0	0	0	**4**

total empirical articles	649	675	664	654	563	456	491	451	463	504	**5570**

**percentage qualitative**	**0.3**	**0.0**	**0.0**	**0.0**	**0.4**	**0.0**	**0.0**	**0.0**	**0.0**	**0.0**	**0.1**

Lancet											

qualitative empirical articles	0	2	2	1	0	0	0	2	1	0	**8**

total empirical articles	931	823	837	881	922	850	738	656	567	600	**7805**

**percentage qualitative**	**0.0**	**0.2**	**0.2**	**0.1**	**0.0**	**0.0**	**0.0**	**0.3**	**0.2**	**0.0**	**0.1**

NEJM											

qualitative empirical articles	0	0	0	0	0	0	0	0	0	0	**0**

total empirical articles	465	448	446	461	508	634	636	611	615	610	**5434**

**percentage qualitative**	**0.0**	**0.0**	**0.0**	**0.0**	**0.0**	**0.0**	**0.0**	**0.0**	**0.0**	**0.0**	**0.0**

PLoS Medicine											

qualitative empirical articles	---	---	---	---	---	0	0	0	1	1	**2**

total empirical articles						42	189	288	222	179	**920**

**percentage qualitative**						**0.0**	**0.0**	**0.0**	**0.5**	**0.6**	**0.2**

**Table 4:** Number/percentage of qualitative articles published in top health services & policy research journals, 1999 to 2008

Journal	1999	2000	2001	2002	2003	2004	2005	2006	2007	2008	Total
Am J Manag Care											

qualitative empirical articles	1	2	1	3	1	2	1	2	2	0	**15**

total empirical articles	124	178	140	164	86	149	140	113	108	116	**1318**

**percentage qualitative**	**0.8**	**1.1**	**0.7**	**1.8**	**1.2**	**1.3**	**0.7**	**1.8**	**1.9**	**0.0**	**1.1**

Health Affairs											

qualitative empirical articles	1	2	1	5	2	2	0	4	3	0	**20**

total empirical articles	133	145	153	182	172	227	251	220	218	178	**1879**

**percentage qualitative**	**0.8**	**1.4**	**0.7**	**2.7**	**1.2**	**0.9**	**0.0**	**1.8**	**1.4**	**0.0**	**1.1**

Health Econ											

qualitative empirical articles	0	0	1	0	1	1	0	0	2	1	**6**

total empirical articles	56	58	57	56	78	87	109	92	86	84	**763**

**percentage qualitative**	**0.0**	**0.0**	**1.8**	**0.0**	**1.3**	**1.1**	**0.0**	**0.0**	**2.3**	**1.2**	**0.8**

Health Serv Res											

qualitative empirical articles	0	0	3	2	0	2	3	5	2	0	**17**

total empirical articles	72	82	74	83	93	110	107	115	126	101	**963**

**percentage qualitative**	**0.0**	**0.0**	**4.1**	**2.4**	**0.0**	**1.8**	**2.8**	**4.3**	**1.6**	**0.0**	**1.8**

J Health Econ											

qualitative empirical articles	0	0	0	0	0	0	0	0	0	0	**0**

total empirical articles	38	51	52	54	53	55	57	60	59	104	**583**

**percentage qualitative**	**0.0**	**0.0**	**0.0**	**0.0**	**0.0**	**0.0**	**0.0**	**0.0**	**0.0**	**0.0**	**0.0**

Med Dec Mak											

qualitative empirical articles	1	0	2	0	1	2	0	2	3	1	**12**

total empirical articles	52	53	54	61	48	58	57	49	64	82	**578**

**percentage qualitative**	**1.9**	**0.0**	**3.7**	**0.0**	**2.1**	**3.4**	**0.0**	**4.1**	**4.7**	**1.2**	**2.1**

Medical Care											

qualitative empirical articles	0	0	3	3	3	1	1	1	1	1	**14**

total empirical articles	180	129	129	167	151	161	175	191	184	183	**1650**

**percentage qualitative**	**0.0**	**0.0**	**2.3**	**1.8**	**2.0**	**0.6**	**0.6**	**0.5**	**0.5**	**0.5**	**0.8**

Milbank Q											

qualitative empirical articles	0	1	0	0	0	1	0	1	0	2	**5**

total empirical articles	21	19	20	23	19	19	29	21	20	22	**213**

**percentage qualitative**	**0.0**	**5.3**	**0.0**	**0.0**	**0.0**	**5.3**	**0.0**	**4.8**	**0.0**	**9.1**	**2.3**

Qual Saf Health Care											

qualitative empirical articles	---	---	---	2	1	6	7	8	5	10	**39**

total empirical articles				66	88	99	102	95	80	82	**612**

**percentage qualitative**				**3.0**	**1.1**	**6.1**	**6.9**	**8.4**	**6.3**	**12.2**	**6.4**

Value Health											

qualitative empirical articles	---	0	0	1	1	0	1	0	0	1	**4**

total empirical articles		12	25	30	45	52	50	46	62	152	**474**

**percentage qualitative**		**0.0**	**0.0**	**3.3**	**2.2**	**0.0**	**2.0**	**0.0**	**0.0**	**0.7**	**0.8**

The total number of qualitative studies published in health services and policy research journals from 1999 to 2008 ranged from 0 in the *Journal of Health Economics *to 39 in *Quality & Safety in Health Care*. The percentage of qualitative studies in health services and policy research journals over this period ranged from 0.0% to 6.4%. No trends in the number of qualitative studies published yearly were apparent.

Table 5 outlines the findings of the validity check, comparing the number of qualitative and total research studies identified by literature search and by tables of contents search in a sample of eligible journals. In both health services and policy research journals examined, there were minimal differences in the number of qualitative and non-qualitative studies identified by the literature and tables of contents searches. For one general medical journal, the table of contents search retrieved more qualitative studies. For both general medical journals, the literature search retrieved more non-qualitative studies compared with the table of contents search.

**Table 5:** Comparison of results achieved by literature and tables of contents searches

Journal	Empirical Articles (qualitative, non-qualitative, ratio)	
	
	Literature search	Tables of contents search
General Medical		

CMAJ 2001	2	2
	221	130
	0.90	1.54

BMJ 2006	1	6
	533	429
	0.19	1.41

Health Services		

Medical Care 2001	3	3
	126	124
	2.38	2.42

Health Affairs 2006	4	6
	216	215
	1.85	2.79

## Discussion

This study found that very few qualitative studies were published in 20 high impact general medical and health services and policy research journals relative to non-qualitative research, and publishing rates of qualitative studies in these journals remained consistently low over the period from 1999 to 2008.

Our findings based on a decade of published research in general medical and health services and policy research journals are similar to those of one study that investigated qualitative research publication rates which reported that 0.6% of studies published in 170 general medical, mental health and nursing journals during the year 2000 were qualitative [15]. Our findings differ from those reported by Weiner et al. in a ten year scan of nine health services and policy research journals from 1998 to 2008, which found that 9% of research articles were qualitative [22]. However, their purpose and methods differed from ours. They focused on the extent to which health services researchers used qualitative methods and for what purpose so they identified qualitative articles and extracted information about the type of qualitative design and how qualitative methods were reported. With respect to methods, it is unclear how they assembled a bibliographic library of all articles published in the nine journals during the specified time period. They sampled the nine journals from those considered important in a survey of health administration faculty in American business schools, whereas we sampled based on several measures of impact. As a result we reviewed the content of three of the nine health services and policy research they examined. In addition we examined general medical journals. Eligibility criteria also differed. Weiner et al. included case studies using both qualitative and quantitative data collection methods (21% of the qualitative studies they identified), and quantitative surveys, whereas we did not. Peripherally relevant research developed optimal search strategies for identifying qualitative research in the nursing [23] and breastfeeding [19] literature, and examined the methods used in studies published in two qualitative research journals [24]. Hence, our findings that consistently few qualitative research studies are published in prominent health care journals are current and unique.

This study is based on the premise that qualitative data are important in health care decision making. While there is no definitive evidence to support this assertion, there are several examples of ways in which qualitative information can be used to improve the quality of health care delivery [8-14]. Still, qualitative research may be considered a "second class citizen" by users unfamiliar with its philosophy and methods [25,26]. It is notable that many general medical and health services and policy research journals devote considerable space and attention to topical issues in the form of commentaries which, despite often being authored by recognized experts, are largely based on anecdotes or opinion, while rigorously conducted qualitative research is not routinely published.

One study found that health professionals believe qualitative research lacks scientific accuracy [27]. If such views about qualitative research are widespread, this may be contributing to the low publication rates in high impact journals demonstrated by this research study. What would constitute definitive evidence of the impact and need for qualitative research, such that it would convince different users of its validity? What is the relevance of qualitative research to users when delivered in various formats. Interviews with different types of health professionals could explore these issues to generate greater understanding about whether and how they consider and use qualitative research. For example, meta-synthesis is an emerging means by which to integrate qualitative research on a common topic into what might be considered strong evidence for decision making [28,29]. At the same time, it is acknowledged that qualitative research might be held in higher regard if qualitative studies were of consistently higher quality, and agreement was established, even among trained qualitative researchers, on various approaches and methods [30]. While guidance is available for conducting and appraising qualitative research [25,31] other forms of education may be required to inform different users about the nature and applications of qualitative research. It is not clear whether journal editors and reviewers use these criteria, or even whether and how the journals referred to in this study accommodate qualitative research by accepting its submission, providing authorship guidelines for qualitative studies, including individuals with qualitative expertise on editorial boards, or training reviewers or providing them with tools to evaluate qualitative submissions [26,32]. BMJ appeared to have a small spike in the number of qualitative articles published. Interviews with journal editors may provide insight into policies and processes that influence whether and how qualitative research is considered and published. At the same time it would be useful to explore the role that qualitative researchers play in qualitative research publishing trends, including the decisions they make with respect to where they submit manuscripts based on qualitative research, and whether such research is targeted to specialty journals rather then general medical and health services and policy research journals.

The accuracy of these data are limited by the capacity to execute searches in MEDLINE that distinguish empirical research from other publication types. We found that this was mainly true for non-qualitative studies. Validation checking by searching tables of contents showed that the number of non-qualitative studies was inflated in MEDLINE searches, so despite extremely low ratios of qualitative to non-qualitative studies reported here, they may still be somewhat over-estimated. However, the limitation likely applies to each journal, and the overall intent of the study was to explore the degree to which qualitative research is published and whether this has increased over time, rather than generating an accurate statistic. Moreover, the actual number of qualitative articles identified in MEDLINE matched, or was quite similar to the number identified in tables of contents searching, which confirms the paucity of qualitative studies published in high impact general medical and health services and policy research journals. Interpretation of the implications of these data may be limited by the calculation of the journal impact factor metric. While much debated, this has been shown to be an accurate statistic [18]. However, to alleviate any concerns, top-ranked journals were selected from among those most frequently represented across four impact ranking systems. It may be that there are fewer qualitative compared with quantitative researchers. Even if this were so, the number of qualitative articles published in major health care journals appears to be so low that there are likely other contributing factors. It might not be the mandate of general medical or health services and policy research journals to publish qualitative research, but journal publication policies were not examined.

## Conclusions

Although qualitative research has the potential to inform and improve health care decisions, this analysis suggests that it is rarely published in high impact general medical and health services and policy research journals. The factors that contribute to this persistent marginalization need to be explored. More insight on a variety of users knowledge of, and views on the utility of qualitative research is needed to better understand how its appropriate use could be expanded.

## Competing interests

The authors declare that they have no competing interests.

## Authors' contributions

Both ARG and MJD conceptualized and planned this research study, collected and analyzed data, summarized and interpreted data, prepared the manuscript, and approved this final version.

## Pre-publication history

The pre-publication history for this paper can be accessed here:

http://www.biomedcentral.com/1472-6963/11/268/prepub
